# Administration of Iranian Propolis attenuates oxidative stress and blood glucose in type II diabetic patients: a randomized, double-blind, placebo-controlled, clinical trial

**DOI:** 10.22088/cjim.10.1.48

**Published:** 2019

**Authors:** Sepideh Hesami, Sima Hashemipour, Mohammad Reza Shiri-Shahsavar, Yaghob Koushan, Hossein Khadem Haghighian

**Affiliations:** 1 Department of Nutrition, School of Health, Qazvin University of Medical Sciences, Qazvin, Iran; 2 Metabolic Diseases Research Center, Qazvin University of Medical Sciences, Qazvin, Iran; 3 Medical Plant Research Center of Islamic Azad University of Tabriz, Researcher in Traditional Medicine, Tabriz, Iran

**Keywords:** Propolis, Type 2 diabetes mellitus, Fructosamine, Catalase activity, Oxidized-LDL

## Abstract

**Background:**

Hyperglycemia in diabetic people resulted in oxidative conditions. Propolis is the third most important component of bee products which has various functional properties such as anti-oxidant due to its components. The aim of this study was to evaluate the propolis effect on fructosamine level, the catalase activity, and the level of oxidized LDL changes in type

2 diabetic patients.

**Methods:**

In this double-blind, randomized controlled trial study, 62 type 2 diabetic patients, attending Endocrine clinic in Velayat Hospital (Qazvin, Iran) in 2017, were randomly assigned to one of intervention and placebo (n=31) groups. Participants in intervention group took propolis capsule (500 mg) 3 times a day and those in placebo group took placebo capsules for 8-week. Fructosamine level, catalase activity and the level of oxidized-LDL were measured at the baseline and at the end of the study. Statistical analysis was performed using SPSS software.

**Results:**

At the end of the study, significant differences were seen within groups and in-between groups. In Propolis group compared to the placebo, fructosamine (p<0.05), and the level of oxidized LDL (p<0.05) decreased, and catalase activity (p<0.05) improved. However, there were no significant changes in the placebo group at the end of trial.

**Conclusion:**

Eight weeks intake of propolis as a supplement in type II diabetic patients could improve anti-oxidant defense and decline production of hyperglycemia induced products such as fructosamine.

Diabetes mellitus, an endocrine metabolic disease, is a leading global health emergency and is associated with serious complications contributing to high morbidity and mortality rates estimated to cost billion dollars per year. It was estimated that in 2015 415 million adults were living with diabetes worldwide, which 90-95% were living with type 2 diabetes mellitus (T2DM) (1). T2DM considerably is attributed to macro and microvascular complications, and comorbidities such as cardiovascular disease; which many of are associated with poor glycemic control (2). Hyperglycemia increases the production of reactive oxygen species (ROS) and also diminishes antioxidative mechanisms by glycation of the scavenging enzymes (3). Accordingly, it is estimated that the down regulation of ROS generation may have an essential role in controlling diabetic complications (4).

Fortunately, the various antioxidant defense systems to remove ROS have been well built in the human body. In other words, various substances containing the non-enzymatic chemicals such as superoxide dismutase (SOD), glutathione peroxidase (GPX), catalase (CAT), vitamin C and E, beta-carotene, and glutathione act as the antioxidant enzyme in the body (5, 6). Catalase, one of the enzymes of the biological antioxidative system, removes hydrogen peroxide, H2O2, by converting it to water and oxygen. H2O2, a most stable forms of ROS might be generated by both exogenous and endogenous factors (7).

Glycation is identified as a detrimental phenomenon in diabetic complications and in association with oxidative stress seems to be detrimental too (8). One of the underlying results of hyperglycemia is the excessive non-enzymatic glycation of the two main proteins: hemoglobin and albumin, known as HbA1c and fructosamine (8, 9). Both fructosamine and HbA1c are used for assessment of glycemic control. However, long half-life of glycated hemoglobin (6–8 weeks), reflects glycemic control over a longer period of time versus fructosamine with a shorter half-life (only 2–3 weeks), has a greater sensitivity to the rapid glucose changes (10). Today there are varieties of natural products which used in treating a large array of systemic diseases. Propolis a non-toxic resinous natural substance was made by bees for building and preserving their hives, by killing pathogens, shielding the honeycomb from the rain. Also, propolis due to its adhesive feature prevents foreign guests entering the hive (11). The biological activity of propolis is mostly related to flavonoids and hydroxycinnamic acid contents (12). Research has shown that flavonoid contents of propolis are dependent on the environmental condition, the site of collection, the origin, and type of plant pollen and species of bees that produced it. Antimicrobial, anticancer, antifungal, antiviral and anti-inflammatory properties ascribe to propolis (12, 13). According to the studies that were conducted in diabetic models, propolis administration has potential to ameliorating antioxidant capacity, by increasing antioxidant enzyme level, and could reduce blood glucose levels which could alleviate diabetic symptoms and result in better diabetic control (14-16). Due to the high cost of prevention and treatment of this disorder, the aim of this study was to evaluate the effect of propolis supplementation on fructosamine level, the catalase activity, and the level of oxidized LDL changes to prove propolis beneficial effects as a supplement on diabetic condition.

## Methods

**Participants:** This double-blind randomized controlled clinical trial was conducted in Velayat Hospital (Qazvin, Iran) on patients with type 2 diabetes. The inclusion criteria were willing to contribute, aged 30-55 years with a medium physical activity (according to a valid and reliable version of the International Physical Activity Questionnaire (IPAQ) in Iran (17)), that did not change their treatment methods and their medications at least in the last two months. Patients were excluded by each of the following conditions: insulin treatment, having diabetes over ten years, pregnancy and lactation, hospitalization during the study, patients with severe renal and hepatic disorders, any acute illness that may affect the study (cardiovascular, pulmonary, kidney, and cancer), change in dose of blood glucose lowering drugs, changes in diet or physical activity levels, dietary supplement consumption two month before, allergy, smoking or alcohol consumption and the occurrence of any side effects due to the intervention.

The protocol of this study was approved by the Ethics Committee of the Qazvin University of Medical Sciences, Qazvin, Iran, and was recorded in the Iranian Registry of Clinical Trials website with a code of IRCT2017041019669N4.

**Sample Size and setting:** According to the GPx factor that was reported in Afsharpour et al.’s study (18), 22 persons were estimated in each group. If the mean and standard deviation of the GPx at the base and the end of intervention were 43±5.2 and 51±7.2 respectively, and by β = 0.2 and α=0.05 assumption in the sample size estimation formula mentioned below, 22 persons were calculated. By considering the probability of 35 percent sample size dropout, 30 persons were selected for each group. 

N= [(Z1-α/2 + Z1-β) 2 (SD12+SD22)] /∆2

**Study Design:** Sixty-two patients (men & women) who referred, were matched by age, gender, and weight, and then randomly assigned to two groups. Windows based software which had been designed to use and allocate number by utilizing a randomized number table was used for number assignment in a random way to each subject. In this method, the investigator and all participants were blinded to treatment. 

The treatment group (n=31) received 1500 mg propolis (500 mg capsule 3 times a day) and the placebo group (n=31) received matching placebo for 60 days. The duration of this study was built on our previous study (18).

Each propolis capsule contained powdered propolis, which originated from Alamut, Qazvin. Propolis was powdered and encapsulated by a Traditional-Medicine researcher at the Azad University of Tabriz, Tabriz, Iran. Placebo capsules contained wheat flour. All capsules had an identical appearance with a specific identifier code in which the investigator and patients, both were blinded until the end of supplementation. The distribution was according to allocation codes after randomization. To ensure blindness, capsules allocation was performed by a trained investigator who did not involve in this trial.

In a face to face interview, demographic data and food recall questionnaires for 3 non-sequential days were collected by a trained nutritionist.

Patient’s weight in minimally clothed was measured with Seca scales and their height was measured in a standing position and without shoes with a fixed‐to‐wall tape meter. After that, BMI (body mass index) was calculated in kilograms per square meter.

**Ethical considerations:** This study was registered at the Ethics Committee of Qazvin University of Medical Sciences that vouched for its ethical considerations Ir.qums.rec.1396.410. At the beginning of the study, the goals and method of the study were fully explained to the patients. They were assured that their medical history and identity would be confined and the possibility of stopping cooperation at any time as they wish. They volunteered and those who agreed and qualified completed a written informed consent.

**Laboratory analysis:** Fasting blood samples were collected before and after supplementation. Hemoglobin tests were performed on the same day. Then with a refrigerated centrifuge, at 4° C and 3000 rpm, the plasma was separated and stored at -70° C for further tests. Fructosamine was assessed in the method of calorimetric enzymatic using commercial kits (Diazyme kit, USA). Measurement of catalase activity was performed by USA Cayman commercial kit. Measurements of oxidized LDL were performed using ELISA kit purchased from Mercodia Sweden.

**Statistical analysis:** The data were analyzed using SPSS 20 for Windows software. All data were presented as mean±SD and were checked for normality by the Kolmogorov–Smirnov test. Due to the normal distribution of variables, the paired sample t-test and the independent sample t-test were applied to analyze differences in variables within and between groups, respectively. The p<0.05 was considered statistically significant.

## Results

**Figure 1:**
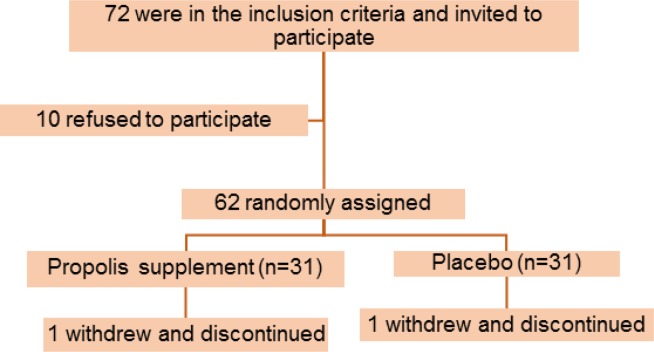
Flowchart of patients' enrolment

Seventy-two patients were enrolled in this study, however, only sixty-two were qualified for the intervention. Two patients from the propolis group and the placebo group dropped out because of personal reasons. The final analysis was done on the subjects who finished the study (figure1).

There were no statistical differences in baseline characteristics of participants between the two groups. The mean age of all participants was 50.43±7.27 years. All the characteristics data are listed in table1. 

**Table 1:** Demographic characteristics of participants at baseline

**Characteristics**		**Propolis(n=30)** **Mean±SD**	**Placebo(n=30)** **Mean±SD**	**p-value**
Age (year)		51.81 ± 6.35	49.05 ± 8.2	0.24
Weight (kg)	Baseline	68.2 ± 9.7	70.76 ±11.7	0.63
	End	68 ± 9.04	71.5 ±11.84	0.42
BMI(kg/m^2^)	Baseline	26.78 ± 3.01	26.74±3.7	0.81
	End	26.7 ± 2.8	27.01 ± 3.7	0.62
Metformin dose		1518.17±329.2	1502.26±410.91	0.91
Diabetes duration (year)		5.47 ± 3.6	5.38 ± 3.1	0.9

Propolis supplementation, compared to the placebo, significantly and statistically decreased the fructosamine (p<0.03), and the level of oxidized LDL (p<0.04), and increased the catalase activity (p<0.05). Within groups, analysis indicated statistical decrease on the fructosamine and the level of oxidized LDL, and statistical increase significance in the catalase activity after intervention in the Propolis group (p<0.05). There were no significant statistical changes in the placebo group. No side effects of the treatment with Propolis were reported during the study. Statistical data are shown in table 2.

**Table 2:** Effect of propolis on the level of fructosamine, catalase activity and oxidized-LDL in two groups

**Variables**		**Mean±SD** **Propolis(n=30)**	**Mean±SD** **Placebo(n=30)**	**P1**
Fructosamine (μmol)	Baseline	391.19±101.82	400.9±87	0.611
	End	336.477±97.21	402.43±103.75	0.019
	P2	0.023	0.847	
Catalase Activity (U/ml)	Baseline	68.35±22.36	72.58±24.36	0.601
	End	83.06±27.37	70.78 ±23.05	0.041
	P2	0.031	0.759	
Oxidized LDL (mU/l)	Baseline	14.43±4.01	13.9±3.67	0.54
	End	9.71±3.24	12.94±3.09	0.031
	P2	0.004	0.428	

## Discussions

Insulin resistance or deficiency of insulin secretion in diabetic patients type 2 cause chronic hyperglycemia and impaired carbohydrates, lipids, and protein metabolism and ultimately promote the generation of reactive oxygen species and oxidative stress (19,20). Hyperglycemia has been seen as the leading cause of diabetic cardiomyopathy by activation of different mechanisms leading to oxidative stress (21).

Several antioxidants including vitamins and supplements as well as flavonoid compounds have been investigated for DM oxidative stress treatment, which has demonstrated a beneficial effect in DM patients (22). For example, Jarouliya et al. demonstrated that Spirulina due to its antioxidant properties not only improved blood glucose levels, but also oxidative markers (23). Furthermore, ROS production in diabetes may activate the MAPK (mitogen-activated protein kinases) signaling cascade, the main component of the proapoptotic signaling pathway, induced by diabetes mellitus can have destructive effects on cellular function. Results in Kadry M. Sadek et al. study revealed that Spirulina has a significant impact on MAPK activity (24). The propolis chemical composition, depending on the plant species from which the bees collect the exudates is highly variable; therefore, geographic location is a major determinant. The most widely used propolis contains mainly phenolic: ﬂavonoids aglycone, aromatic acids, and their esters (25). The propolis biological action is taken from its active constituents including flavonoids with antioxidants (26). The present study investigated the effect of Iranian Propolis on fructosamine, the catalase activity and changes in oxidized LDL level in type II diabetes.

Reports have demonstrated that concentrations of serum advanced glycation end-products (AGEs) elevate in healthy individuals with higher ages, and these were associated with elevated levels of oxidative stress and inflammation (27). Chronic hyperglycemia in uncontrolled diabetes increases AGE formation which will also lead to increased RAGE signaling (receptors for AGEs), oxidative stress and inflammation. Increased AGE may lead to oxidative stress and, vice versa (28, 29).

Unfortunately, there is a point that oxidative stress is a key mechanism for albumin glycation in individuals without diabetes (30). Accumulating data has shown that serum fructosamine is prospectively associated with morbidity-mortality risks of cardiovascular disease in both diabetic and non-diabetic patients (30, 31). Fuliang et al. demonstrated that fructosamine levels decreased in rats fed with ethanol extract of Propolis (EEP). They observed that EEP reduced plasma glucose levels, suppressed oxidative stress, and reduced oxidants in diabetic rats (32). Since this regent is the first product in the glycation procedure, it supports the observation of the antiglycation effect of EEP. Another recent study reported the chelating property of propolis can play an important role in the anti-glycation activity of propolis (33, 34). The small minority of studies investigated the effects of propolis on fructosamine and of these, there is no single study that has been done in human, however, our results in confirmation of animal studies, showed that 8-week propolis supplementation led to lower fructosamine formation in diabetic people.

It has been confirmed that the mitochondrial catalase overexpression can reduce the prevalence of arteriosclerosis and cataract in mice, and also protects against cardiovascular dysfunctions and injuries in human (35, 36). In our study, catalase activity remarkably was enhanced in the propolis group in comparison to baseline and the placebo group. This outcome is consistent with the properties that were attributed to the propolis chemical composition as well as studies on antioxidant capacity changes due to propolis consumption. At the end of a 18-week study, recruited T2DM patients who were treated by Chinese propolis (900 mg/day) serum GSH, ﬂavonoids, and polyphenols signiﬁcantly increased, and serum lactate dehydrogenase activity was signiﬁcantly reduced, however, no signiﬁcant difference was found between the groups in serum glucose, glycosylated hemoglobin, insulin (16). Another study reported that the levels of glutathione peroxidase and catalase (antioxidant enzymes) increased in rats fed with EEP treated with sodium fluoride in comparison with the control group (37). 

Oxidative stress by excessive generation of prooxidant species, as well as deficiency of antioxidant defense mechanisms result in damage to different cellular components. The most susceptible targets of oxidation are lipids and plasma oxidized LDL (ox-LDL) playing a significant role in atherosclerosis development (38, 39). Several studies in adults demonstrated significant correlations between high circulating ox-LDL levels and CVDs, diabetes and metabolic syndrome prevalence (40).

The powerful antioxidant potential of propolis is active against oxidative stress and may induce a positive effect on diabetic metabolic complications. Verónica Mujica et al. have shown that propolis leads to thiobarbituric acid reactive substances (TBARS) levels dwindling which are formed as a byproduct of lipid peroxidation and increase glutathione (GSH) levels, a powerful antioxidant that protects the important cellular components against free radicals. Results suggested that the inhibitory effect on lipid peroxidation of propolis reduces the production of oxyger free radicles and decrease oxidative stress (41). Another study reported that propolis supplementation for 12 weeks can result in glycemic and some lipid levels reduction in type 2 diabetes patients (42). 

Serum cholesterol, triglycerides, LDLc, VLDL, atherogenic and atherogenic parameters significantly declined and serum HDLc increaset in intoxicated male albino mice that were treated by aqueous extract of Libyan propolis. It can be concluded propolis had hypolipidemic and antiatherogenic effects in mice (43). Results of treatment of both types of propolis (Chinese and Brazilian) by Hongzhuan Xuan study significantly indicated increased cell viability and attenuated apoptosis rate. This study also showed that both types of Propolis inhibited the effect of ox-LDL in human umbilical vein endothelial cells on ROS generation as well as the subsequent MMP collapse, and NF-𝜅Bp65 activation (44). Similar to the articles listed above the final analysis of our data suggested that 8-weeks propolis treatment has the power to reduce ox-LDL in diabetic people.

The results of the present study demonstrated that Iranian propolis treatment significantly diminished fructosamine and oxidized-LDL after 8-week supplementation and also catalase activity improved by supplementation in the propolis group. Nevertheless, due to budget limitations, we were not able to consider a larger sample size, different doses, longer duration, and considering other groups (such as an antioxidant group) to explain the clinical relevance of finding data. In conclusion, propolis has a potential to improve antioxidant capacity (catalase activity) and alleviate AGEs products and lipid oxidation in DM patients. 
